# Chinese Herbal Medicines Compared with N-Acetylcysteine for the Treatment of Idiopathic Pulmonary Fibrosis: A Systematic Review of Randomized Controlled Trials

**DOI:** 10.1155/2019/5170638

**Published:** 2019-06-13

**Authors:** Jing Guo, Bin Li, Wenbin Wu, Zhichao Wang, Fei Wang, Taipin Guo

**Affiliations:** ^1^Hospital of Chengdu University of Traditional Chinese Medicine, Chengdu, Sichuan 610072, China; ^2^School of Acupuncture, Moxibustion, Tuina and Rehabilitation, Yunnan University of Chinese Medicine, Kunming 650500, China

## Abstract

**Background:**

Idiopathic pulmonary fibrosis (IPF) is a major global health problem. The prevalence of the disease appears to be increasing. There is no curative therapy for IPF except lung transplantation. Chinese herbal medicines (CHMs) are showing promise for treatment of IPF. However, their effectiveness and safety are still unclear and deserve further investigation. The aim of this systematic review is to access the efficacy and safety of CHMs in treating IPF.

**Methods:**

The protocol of this review is registered at PROSPERO. We searched seven main databases for randomized clinical trials (RCTs) on CHMs for IPF from their inception to June 4, 2018. The methodological quality of RCTs was assessed using the Cochrane risk of bias tool. All trials included were analyzed according to the criteria of the Cochrane Handbook. Review Manager 5.3, R-3.5.2 software, and Grade pro GDT web solution were used for data synthesis and analysis.

**Results:**

Thirteen randomized clinical trials enrolling 733 patients were included. All trials included had clear outcome indicators. The methodological quality of included trials was generally “poor.” Few trials reported methods of randomization. One trial on Xuefu-zhuyu capsule assessed rate of acute exacerbation and mortality after treatment for 72 weeks and found no statistically significant difference between two groups. This meta-analysis demonstrated a significant improvement in QOL of IPF patients when CHMs was applied or combined with conventional medicine treatment. 6MWT was significantly improved in IPF patients after using CHMs or combined with conventional medicine treatment. CHMs treatment also had a certain improvement in TLC and DLCO, but the effect on FVC was not significant. Besides, CHMs failed to provide benefits in terms of PaO_2_. The reported adverse events were not obvious and severe.

**Conclusions:**

Some CHMs seem effective and safe as alternative remedies for patients with IPF, suggesting that further study of CHMs in the treatment of IPF is warranted. Although this systematic review suggests that CHMs may have positive effect on quality of life, 6-minute walk test distance, and lung function (TLC, DLOC%) and seem to be relatively safe during the course of treatment, the results must be treated with great caution because of the methodological flaws of the included trials. Long-term and high-quality trials are needed in the future to provide clear evidence for the use of CHMs.

## 1. Introduction

Idiopathic pulmonary fibrosis (IPF) is a chronic, progressive, and irreversible pulmonary disease of unknown etiology that usually affects older adults and is limited to the lungs. Adults with chronic labor dyspnea due to unexplained causes, and the clinical manifestations of cough, lung bases Velcro rales, and clubbing of fingers should consider the possibility of IPF. IPF is more common in men and is rare in people younger than 60-70 age group. IPF occurs worldwide. The incidence of IPF increases with age, and most patients have a history of smoking [1]. The prevalence and incidence of IPF are increasing year by year, and the worldwide incidence rate is similar to that of several malignancies, such as hepatic, testicular, and cervical cancer [2]. Because of its rapid progress, poor prognosis, and high mortality, IPF is called a difficult lung disease. It is a highly morbid and ultimately fatal disease with a median survival estimated at 3-5 years from the time of diagnosis [3–8]. The survival outcome is similar to that of malignant tumors, which seriously threatens the health of patients [9, 10]. Increasing rates of hospital admissions and mortality due to IPF also suggest an increasing burden of disease [11, 12].

Currently, in addition to lung transplantation, there is no effective drug or therapeutics for IPF in the field of western medicine [9]. Unfortunately, only a small number of patients can receive lung transplantation because of the complexity of surgery and postoperative treatment and management, and the restricted supply of donor organs. There is currently no consensus on the optimal treatment of IPF. There is no evidence to prove which drug can effectively treat the disease, and only a few studies suggest that some drugs may be beneficial for IPF treatment. Therefore, the prevention and treatment of idiopathic pulmonary fibrosis has become a problem to be solved in modern medical research. Historically, idiopathic pulmonary fibrosis was considered a chronic inflammatory disorder, which gradually progressed to established fibrosis. However, with the deepening of studies on IPF, experts and scholars have realized that anti-inflammatory therapy cannot improve the prognosis of IPF and have reevaluated this concept. Subsequently, a treatment strategy combining hormones and immunosuppressants was shown to increase mortality [13, 14]. According to official reports, the efficacy of corticoids and immunosuppressants currently widely used in clinical practice is not exact, and these drugs are easy to form dependence and have many side effects [1, 9]. The 2011 IPF guideline of ATS/ERS/JRS/ALAT pronounces a strong recommendation against this therapy [9]. It is important to highlight that although drugs recommended for use in the guidelines, such as Pirfenidone and Nintedanib administration, have been shown to delay the decline in lung function, and they are rarely used clinically in China because of their long-term economic costs and significant adverse reactions. As the research continues to deepen, oxidation-antioxidant imbalance is considered to be involved in the pathogenesis of IPF [15]. Antioxidant therapy has been proposed as an effective treatment for IPF [16]. Thus, administration of antioxidants, such as N-acetylcysteine (NAC), may represent a potential therapeutic option for IPF patients. Although the 2015 updated IPF guideline of ATS/ERS/JRS/ALAT recommended against treatment with N-acetylcysteine as conditional, it still pointed out that N-acetylcysteine monotherapy could improve patients' mental health and walking distance, and it was not recommended to terminate treatment for patients who had received NAC monotherapy [1, 17, 18]. Some studies indicated that N-acetylcysteine monotherapy may have beneficial effects in patients with early-stage IPF and delay disease progression [19, 20]. N-acetylcysteine is a biologically reliable antioxidant and antifibrotic drug that is inexpensive, well tolerated, and easily administered orally, that may be beneficial in patients with IPF [21]. Therefore, it is widely used in clinical practice. However, the safety and efficacy of N-acetylcysteine in patients with IPF remains controversial due to the lack of evidence-based evidence.

Currently, Traditional Chinese Medicine (TCM), as an adjuvant therapy to Western medicine, has its unique superiority and efficacy and is still used widely for the treatment of IPF in China. TCM is a potential therapy for IPF due to its advantages of syndrome differentiation for treatment, small adverse reactions, stable curative effect, long duration, overall regulation, and no obvious drug dependence. More and more formulas, herbal compounds, and several types of Chinese herbal medicine extracts were proved to have an effect in preclinical experimental studies of IPF patients in China [22–24]. Moreover, many kinds of Chinese herbs like ligustrazine and its extracts are widely used for IPF patients in China [25]. A universal strategy of CHMs treatment of IPF is to effectively alleviate clinical symptoms, improve the quality of life, and reduce mortality of patients [26–28]. The research results of IPF prevention and treatment by CHMs indicate that it can alleviate the symptoms of cough, expectoration, wheezing, and dyspnea, increase arterial oxygen partial pressure, shorten the course of disease, and improve the quality of life of patients [29]. Therefore, the treatment of IPF with CHMs has become a feasible and superior therapy, providing new ideas for solving the controversial problem of selecting effective drugs. Unfortunately, the overall quality of the systematic review on the treatment of IPF by CHMs is not high at present and this type of meta-analysis is essentially difficult because of the complexity and significant clinical heterogeneity of TCM treatment [30]. Furthermore, due to the small sample size, large differences in intervention measures, and unreasonable selection of control drugs in related studies, the reliable evidence for the efficacy and safety evaluation of CHMs treatment of IPF is still insufficient. In this study, oral N-acetylcysteine was used as the control group, and the evidence-based systematic evaluation method was adopted to strictly evaluate the clinical trial literature of TCM for IPF. The goals of this systematic review are to summarize and analyze the results of all randomized controlled trials (RCTs) employing CHMs in the treatment of IPF and to obtain evidence on the clinical efficacy and safety of CHMs compared with NAC for IPF.

## 2. Materials and Methods

### 2.1. Standard Protocol Registrations

This study has been registered in PROSPERO (http://www.crd.york.ac.uk/PROSPERO), registration number: CRD 42018105348 and the protocol has been published [31, 32]. The procedure of this study is strictly based on Preferred Reporting Items for Systematic Reviews and Meta-Analysis (PRISMA) statement [33].

### 2.2. Database and Search Strategy

The following seven online databases were searched for relevant studies from their inception up to June 4, 2018: Cochrane Library, PubMed, EMBASE, China National Knowledge Infrastructure Database (CNKI), Chinese Biomedical Literature Database (Sino-Med), VIP Chinese Science and Technology Periodical Database (VIP), and Wan Fang Database. The search strategy was based on the guidance of the Cochrane handbook. The following searching terms as abstract terms and MeSH Terms were used individually or combined including (“Traditional Chinese medicine” OR “Chinese medicinal” OR “Chinese medicine” OR “Chinese herbal medicine” OR “decoction” OR “Chinese patent medicine” OR “Chinese medicine preparation” OR “integration of Chinese and Western medicine”) AND (“Idiopathic pulmonary fibrosis” OR “pulmonary fibrosis” OR “pulmonary interstitial fibrosis” OR “Idiopathic pulmonary interstitial fibrosis” OR “IPF” OR “PF”) AND “random*∗*”. The search strategy for PubMed can be found in Appendix S1, and other search strategies can be available through contacting corresponding author. All relevant publications, including dissertation and conference papers, were researched to ensure a comprehensive search. The language is limited to Chinese and English.

### 2.3. Inclusion Criteria

#### 2.3.1. Types of Studies

Only relevant randomized controlled trials (RCTs) were included. Quasirandomized trials were excluded.

#### 2.3.2. Types of Participants

There were no limitations on the patient's gender, age, and the course and severity of the disease. Diagnostic criteria based on the official 2011 idiopathic pulmonary fibrosis ATS/ERS/JRS/ALAT Update Statement [9]. In addition, we also consult the consensus of Chinese experts on IPF diagnosis and treatment formulated by the interstitial pulmonary disease group of the Chinese academy of respiratory medicine in 2016 [34].

#### 2.3.3. Types of Intervention

The experimental group was treated with oral administration Chinese herbal medicine (or combined with western medicine conventional therapy), and the control group was treated with oral administration N-acetylcysteine. The course of treatment in both groups was no less than 12 weeks. The Chinese herbal medicines in this study include decoction, tablet, pill, powder, orally taken paste, sublimed preparation, Chinese herbal compound prescription, single Chinese herbal medicine, and Chinese patent herbal medicine. Both groups could receive the same western medicine routine treatment, including oxygen therapy, infection control, and nutritional support.

#### 2.3.4. Types of Outcome Measures

The primary outcomes were the rate of acute exacerbation; mortality after treatment; health-related quality of life (St. George's Respiratory questionnaire scores).

The secondary outcomes were pulmonary function tests, especially forced vital capacity (FVC), total lung capacity (TLC), and the diffusing capacity of the lungs for carbon monoxide (DLCO); 6-minute walk test distance (6MWT); partial pressure of oxygen in blood (PaO_2_); adverse events. The time endpoint of the above outcomes was not earlier than 12 weeks after the medication.

### 2.4. Exclusion Criteria

(1) The duplicated documents were deleted.

(2) Unrelated papers were excluded, such as animal experiments, reviews, theoretical discussions, experience summaries, and case reports.

(3) The control group in the studies was not treated with N-acetylcysteine.

(4) The drugs were not taken orally in the intervention and control groups.

(5) Articles without original data were excluded.

### 2.5. Data Collection and Extraction

Study screening and data extraction were referred to the Cochrane Handbook for Systematic Reviews of Interventions [35]: (1) the retrieval results were imported into the literature management software of NoteExpress (version:3.2; Beijing Aegean Software Company, Beijing, China); (2) the duplicate literature was eliminated through NoteExpress3.2; (3) through reading the title and abstract, the literature irrelevant to this study was excluded; (4) the full text was read to retain clinical studies that met the inclusion criteria. Two researchers (GJ and LB) extracted the data independently using a self-developed and standard data extraction form. The divergences encountered in the process were resolved by discussing them with another team member (WWB), to determine the final selection of studies by agreement.

Data extraction contents include the following: (1) general information: research ID (the author, year of publication), title, publication status, report sources, and fund support; (2) methodological information: design type, grouping, random sequence generation, allocation concealment, blinding, number lost during followup, selective reporting, sample size calculation, and baseline comparability; (3) participant information: diagnostic criteria, inclusion criteria, exclusion criteria, source of patients, population, sample size, age, gender, and course of disease; (4) intervention information: details of the intervention and control, syndrome differentiation of TCM, dosage form, combination therapy, duration of treatment, and patient followup; (5) outcomes; (6) adverse events.

### 2.6. Assessment of Methodological Quality

The Cochrane risk assessment tool was used for the assessment [36]. Risk of bias was assessed as follows: adequacy of generation of the allocation sequence, allocation concealment, double blinding, incomplete outcome date, selective outcome reporting, followup, and other bias. These domains classified “Yes” if adequate, “No” if not adequate, and “Unclear” if not well described by the authors in such a way that its adequacy is describable.

The two researchers (GJ and LB) independently assessed the risk of bias for included studies. “L,” “H,” and “U” were used as a code for the evaluations of the above bias risks. “L” indicated a low risk of bias, “H” indicated a high risk of bias, and “U” indicated that the risk of bias was unclear. Trials that met all criteria were judged to have a low risk of bias and trials that met none of the criteria were evaluated to have a high risk of bias. The risk of bias in trials with insufficient information was classified as unclear. Disagreements were resolved by discussion and consensus. When necessary, we contacted the authors of the included studies to inquire some missing information.

### 2.7. Data Synthesis and Analysis

Review Manager Software (RevMan, Version 5.3 for windows, The Cochrane Collaboration, Oxford, England) was used to analyze and synthesize the outcomes. Clinical heterogeneity is the primary source of heterogeneity in the systematic reviews of CHMs. Clinical heterogeneity can be derived from the potential factors such as the ingredients and formulations of CHMs [37]. Quantitative synthesis would be done if clinical heterogeneity is not considered by at least two authors in discussion. Continuous variable was described by mean difference (MD), P value, and 95% confidence interval (CI). For dichotomous outcomes, we used the relative risk (RR), with 95% CI and p values, to evaluate the efficacy and safety of CHMs. I^2^ test was used to judge the heterogeneity of meta-analysis. I^2^ value>50% or more was considered as an indication of substantial heterogeneity. If heterogeneity existed in the pooled studies, the data were analyzed using a random effects model. Otherwise, a fixed effect model would be used. If there is significant clinical heterogeneity, the cause of heterogeneity should be explored, and sensitivity analysis or subgroup analysis would be carried out when necessary. Sensitivity analysis was adopted to ensure the robustness of results by eliminating low-quality trials. Subgroup analysis was performed according to the characteristics of the study subjects, such as different types of CHM interventions, treatment duration, and outcome measures. If the data extraction is insufficient, qualitative analysis would be used.

### 2.8. Publication Bias

The publication bias was analyzed using funnel plot when the number of studies included in a meta-analysis is no less than 10. If the number of included studies was less than 10, the Egger's test was applied. The analysis software of Egger's test was R 3.5.2 for Windows.

### 2.9. Quality of Evidence

This study evaluated the evidence according to GRADE standard, which refers to grading of recommendations assessment, development, and evaluation. Many factors that could reduce the quality of evidence were considered, such as limitations in study design, inconsistency of results, indirectness of evidence, inaccuracy, and publication bias. GRADE Pro GDT online software was used to form the summary of findings table (SoF table).

## 3. Results

### 3.1. Selection of Studies

**Figure 1:**
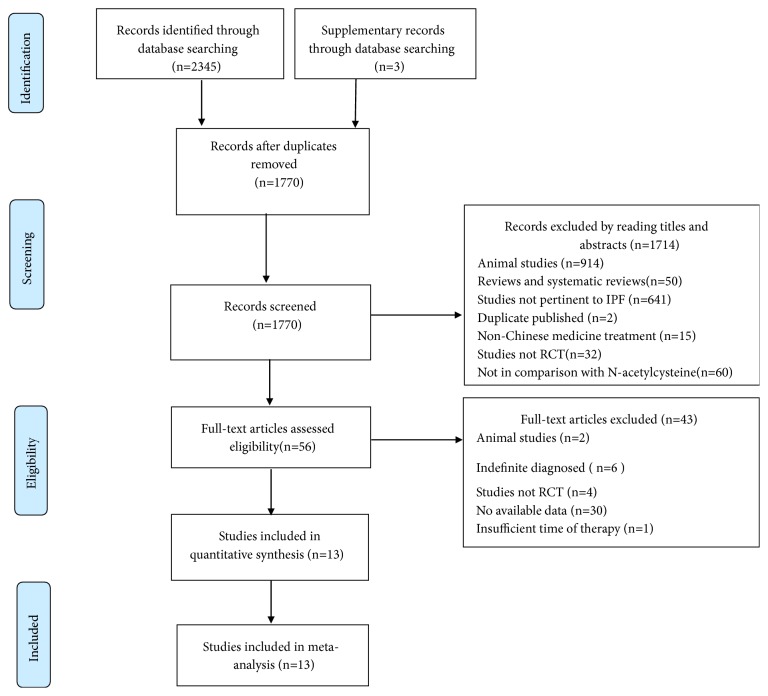
Flowchart of the trial selection process. RCT: randomized controlled trials.

In this study, a total of 2348 studies were retrieved through electronic and manual searches. At the beginning, 578 duplicate studies were removed by using the software of NoteExpress3.2. During the first screening process, 1714 studies were excluded after reading their titles and abstracts. After reviewing the full texts of 56 studies, 43 studies were excluded, and the remaining 13 studies [38–50] were finally included in this study. The search process and study selection are presented by a flow chart (Figure 1).

### 3.2. Characteristics of Studies

Overall, results obtained from 733 IPF patients were selected from 13 RCTs. All the included trials were conducted in China from 2012 to 2018, including ten trials published in the journal [38, 39, 41–47, 50] and three dissertations [40, 48, 49].

Of all the included trials, only one trial [46] did not report the sex ratio of the subjects, and others reporting sex ratios included 302 males and 336 females. Baseline comparisons were made for each study, including sample size, age, gender, the course, and severity of disease. The baseline of the intervention and control groups was consistent.

There are four types of comparison in 13 RCTs. Four trials compared CHMs with NAC in the treatment of IPF. Three trials compared CHMs plus NAC with NAC alone. Six RCTs comparing the efficacy on IPF of CHMs and NAC were identified based on the conventional medicine treatment (CM).

The types of oral administration CHMs were as follows: herbal decoctions were used in ten trials [38–43, 45, 47, 49, 50], capsule was used in one trial [46], pill was used in one trial [48], and granule was used in one trial [44]. Furthermore, all included RCTs in this study have clear outcome indicators. The duration of treatment varied from 12 weeks to 72 weeks. Among them, ten trials lasted for 12 weeks, two trials [45, 50] for 24 weeks, and one trial [46] for 72 weeks. The detailed characteristics of the eligible studies are shown in Table 1.

### 3.3. Risk of Bias in Included Studies

The risk of bias for each study was assessed according to Section 5.1.0 of the Cochrane Handbook for Systematic Reviews of Interventions [35]. Nine studies described the random sequence generation, seven of them used random number table, one of them used computer software, and the other used random lottery, and hence they were evaluated as “low.” The other studies did not report any randomization procedure and were evaluated as “unclear.” One study [46] adopted a blinded approach, and the intervention was designed as a double-blinding, double-simulation with low risk of bias. Other studies did not use blind methods, so risk of bias was assessed as “high.” A total of 10 trials reported loss to followup, the number was small, and the risk of bias was low. The remaining trials did not report whether there was a loss to followup. However, Intention-To-Treat (ITT) was not carried out in all cases of shedding and loss to followup, so incomplete data reporting was possible.

**Figure 2:**
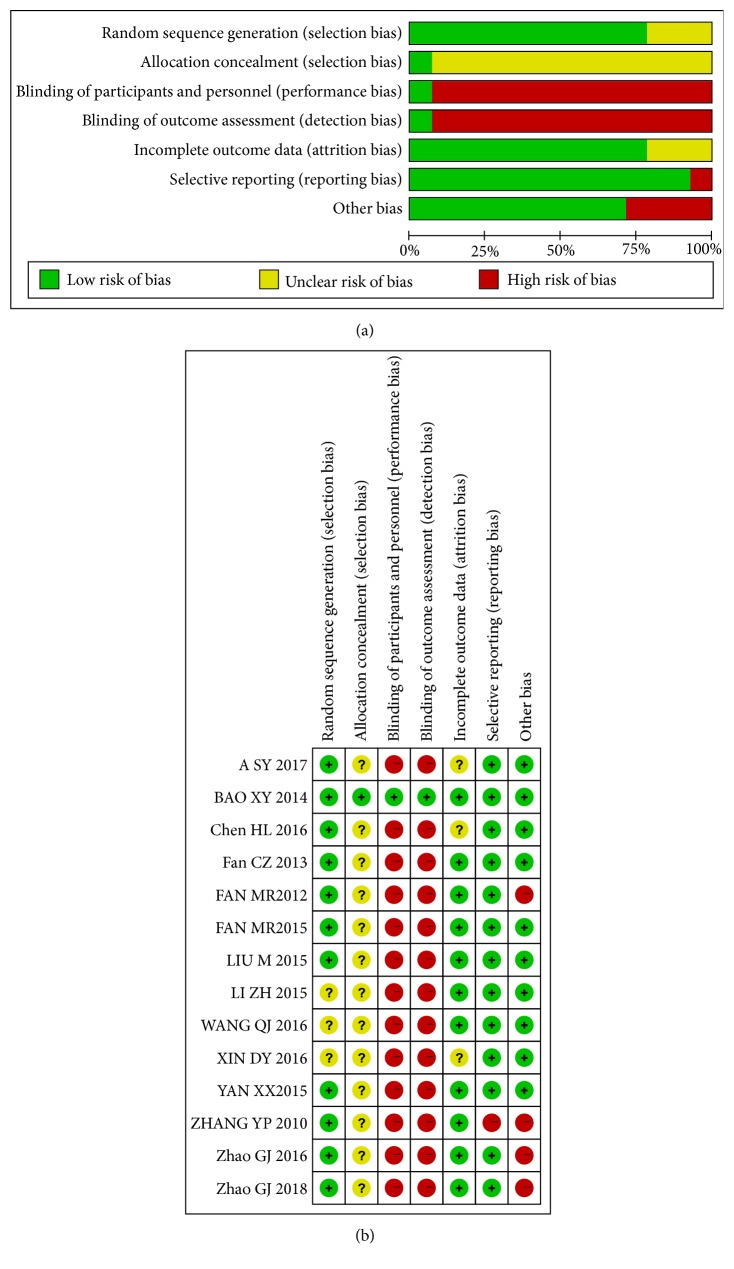
Risk of bias. (a) Risk of bias graph: the authors assessed each risk of bias item presented as percentages across all included studies. (b) Risk of bias summary: the authors judged each risk of bias item for each included study. +: low risk of bias; −: high risk of bias; ?: unclear.

All trials were not registered and could not verify the existence of a selective outcome reporting. The outcome reporting was found to be completely consistent in the results section and methodology section. After discussion, we believe that the risk of bias is low. Of all the RCTs included, only two trials reported sample size estimates. Five trials reported the source of research funding. Methodological quality of the included trials was shown in Figure 2.

### 3.4. Effects of Interventions

#### 3.4.1. Rate of Acute Exacerbation: 72 Weeks

Of the 13 trials included, only one [46] reported the rate of acute exacerbations. The results showed that the acute exacerbation rate was not improved after 72 weeks of treatment with Xuefu-zhuyu capsule plus NAC compared with NAC alone (RR: 0.44,95%CI: [0.04,4.45], P=0.49).

#### 3.4.2. Mortality: 72 Weeks

Only one of all included studies [46] reported mortality from IPF. The results showed that compared with NAC alone, Xuefu-zhuyu capsule combined with NAC did not improve the mortality of patients after 72 weeks (RR: 0.3,95%CI: [0.01,6.84],* P*=0.45).

#### 3.4.3. SGRQ Score

**Figure 3:**
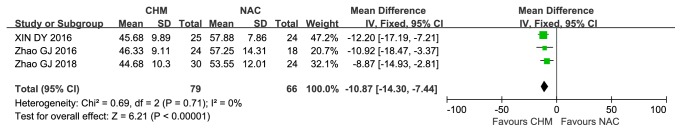
Comparison of Chinese herbal medicines versus N-acetylcysteine, SGRQ.

The quality of life (QOL) in patients with IPF was reported in 6 trials [39–41, 43, 45, 49], which was measured by SGRQ score. Three of the six trials [39–41] reported the control types of CHM versus NAC. Compared with NAC treatment alone (Figure 3), the CHM group Erjia- xiaozheng decoction and Fuzheng-huaxian decoction had a significantly better effect on the SGRQ scores (MD:-10.87, Fixed, 95%CI:[-14.30,-7.44], I^2^=0%,* P*< 0.00001).Then, after 24 weeks of treatment, the combination of CHM Jiawei-xiayuxue decoction plus NAC also had a significantly better effect on the SGRQ scores than NAC alone (MD:-3.48,95%CI:[-6.86, -0.10],* P*=0.04).One trial [43] showed that there was no statistically significant difference between Buxu-tongbi decoction plus CM and NAC plus CM. (MD:-4.36,95%CI:[-18.50, 9.78],* P*=0.55). However, another trial [49] showed that Tongfeiluo-buzongqi decoction plus NAC and CM had a significantly better effect on the SGRQ scores than NAC plus CM (MD:-13.11,95%CI:[-25.46,-0.76], P=0.04).

#### 3.4.4. Lung Function

*Forced Vital Capacity (FVC)*. Three trials [40, 41, 45] reported FVC % pred. Compared with NAC treatment alone, Fuzheng-huaxian decoction had no difference in improving FVC (MD:3.47, Fixed, 95%CI: [-1.78, 8.73], I^2^=0%,* P*=0.20). Jiawei-xiayuxue decoction plus NAC also had no difference in improving FVC compared with NAC treatment alone after 24 weeks of treatment (MD:1.62,95%CI: [-7.66, 10.90],* P*=0.73).

*Total Lung Capacity (TLC). *Eight trials [28, 29, 38, 40, 42, 44, 45] reported TLC% pred. Among them, two trials [38, 40] showed that the CHM group was superior to the NAC group in delaying the TLC decline (MD:5.35, Fixed,95%CI:[0.08,10.62], I^2^=19%,* P*=0.05). In the comparison between Yiqi Tongluo Jiedu decoction and NAC, the results obtained are the same as the overall results (MD:8.70,95%CI:[0.78,16.62],* P*=0.03).Two trials [45, 47] showed that compared with NAC treatment alone, a combination of CHM and NAC had no significant difference in the improvement of TLC. Two trials [42, 44] showed that there was no difference in improving TLC between the CHM plus CM group and the NAC plus CM group (MD: −1.57, Fixed,95%CI: [-5.58, 2.44], I^2^=0%, P=0.58). In addition, two trials [48, 49] on Kangxian granule and Tongfeiluo-buzongqi decoction plus NAC combined with CM were associated with a substantial reduction in the TLC compared with NAC plus CM (MD:5.49, Fixed,95%CI: [2.59, 8.39], I^2^=0%,* P*=0.0002).

*Diffusing Capacity of the Lung for Carbon Monoxide (DLCO). *Totally, ten trials [38, 40–45, 47–49] reported DLCO% pred. The meta-analysis of three trials [38, 40, 41] with CHM vs. NAC showed that there was no statistically significant difference between two groups in improving DLCO (MD:3.79, Fixed,95%CI: [-0.56, 8.13], I^2^=17%,* P*=0.09). CHM+NAC vs. NAC treatment of IPF was tested in two trials [45, 47]. One of the trials [47] showed that there was no significant difference between two groups (MD:5.62,95%CI:[-2.12,13.36],* P*=0.15).In addition, the other trial [45] showed that after treatment for 24 weeks with Jiawei Xiayuxue decoction plus NAC, it was superior to NAC alone in improving DLCO (MD:10.12,95%CI:[0.51,19.73],* P*=0.04).Two trials [42, 44] show that on the basis of CM, Feixiantong decoction and Yangyin Yifei Tongluo pill were significantly better than NAC in improving DLCO (MD:6.51, Fixed,95%CI: [3.01,10.01], I^2^=0%,* P*=0.0003). Two trials [48, 49] showed that CHM plus NAC was significantly better than NAC in improving DLCO on the basis of CM (MD:4.42, Fixed,95%CI:[1.03,7.80], I^2^=0%,* P*=0.01).Specifically, the trial [48] on Kangxian granule was consistent with the overall results (MD:4.50,95%CI:[0.90, 8.10],* P*=0.01).

#### 3.4.5. 6-Minute Walk Test Distance (6MWT)

**Figure 4:**
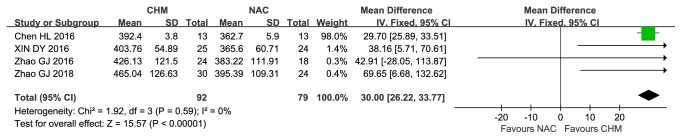
Comparison of Chinese herbal medicines versus N-acetylcysteine, 6WMT.

Nine trials [38–45, 49] reported 6MWT(m). Compared with NAC treatment alone, four trials [38–41] showed that CHM group Fuzheng-huaxian decoction, Erjia-xiaozheng decoction, and Yiqi Tongluo Jiedu decoction were significantly better than NAC alone in improving 6-minute walk test distance (MD:30.00, Fixed,95%CI: [26.22, 33.77], I^2^=0%,* P*<0.00001) (Figure 4). The other trial [45] showed that after 24 weeks of treatment with Jiaiwei-xiayuxue decoction plus NAC, the efficacy in improving 6 MWT was better (MD:16.93,95%CI: [0.45,33.41],* P=*0.04).

**Figure 5:**
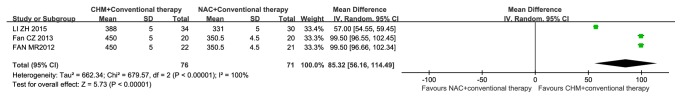
Comparison of Chinese herbal medicines + conventional therapy versus N-acetylcysteine + conventional therapy, 6WMT.

Three trials [42–44] showed that, based on CM (Figure 5), Feixiantong decoction, Yangyin Yifei Tongluo pill, and Buxu -tongbi decoction are significantly better than NAC in improving 6 MWT (MD:85.32, Random,95%CI: [56.16,114.49], I^2^=100%,* P*<0.00001). One trial [49] showed that on the basis of CM, Tongfeiluo-buzongqi decoction combined with NAC was better than NAC in improving 6MWT (MD:109.22,95%CI: [12.41,206.03],* P*=0.03).

#### 3.4.6. Partial Pressure of Oxygen in Blood (PaO_2_)

Three trials [40, 42, 48] reported changes in PaO_2_ (mmHg) between the intervention group and the control group before and after treatment. One of the three trials [40] showed no difference in PaO_2_ changes between the CHM group and the NAC group (MD:1.73,95%CI: [-6.16,9.62],* P*=0.67). Compared with NAC plus CM, no significant difference was found between two groups in one trial [42] on Feixiantong decoction plus CM (MD:0.33,95%CI:-5.17,5.83],* P*=0.91), and the effect of Kangxian granule combined with NAC and CM was significantly better in improving PaO_2_ [48] (MD:9.60,95%CI:[7.51,11.69],* P*<0.00001).

#### 3.4.7. Adverse Events

No obvious adverse events occurred in 13 trials. Adverse events were reported in eight trials [40–43, 46, 48–50]. Of these, five studies [40–43, 48] reported no adverse events occurring in the intervention group and the control group after treatment, and three studies [46, 49, 50] reported the details of adverse events, which included gastrointestinal discomfort, constipation, dry mouth, nausea, dizziness, and drowsiness. Most of the adverse events were not severe. They disappeared without special treatment. One trial [40] showed that the incidence of adverse events treated with Fuzheng-huaxian prescription was lower than NAC alone (MD:0.06,95%CI:[0.00,0.97], P=0.05).One trial [46] showed there was no difference in the incidence of adverse events after 72 weeks of Xuefu-zhuyu capsule plus NAC compared with NAC alone (MD: 1.78, 95% CI: [0.18, 17.80],* P*=0.62). Two trials [39, 49] showed that there was no difference in the incidence of adverse events between the CHM plus NAC group and the NAC group (MD:0.57, Fixed,95%CI: [0.17,1.88], I^2^=0%,* P*=0.36).

### 3.5. Publication Bias

Egger's test and the funnel plot were used to assess the publication bias of the included studies. In this study, the number of included trials in the meta-analysis was less than 10; a funnel plot was not applicable to assess publication bias [51]. Therefore, the Egger linear regression was available for analysis [52]. Among the primary outcomes, only one study reported mortality and the acute exacerbation rate, so the St. George score was applied to analyze publication bias. In the trials reporting the St. George score, there was more than one trial of CHM versus NAC, and the Egger's test results were t= -0.69, P=0.6168, suggesting that there was not major publication bias. Nevertheless, the possibility of publication bias in reporting clinical trial results is always a concern. We conducted comprehensive searches and tried to avoid bias; we found all trials published in Chinese. Almost all studies included reporting positive findings and the trials with negative findings were usually not published. Obviously, the efficacy of CHMs in treating IPF could be exaggerated if only positive findings were published. Therefore, we could not exclude potential publication bias.

## 4. Discussion

This study of the recent randomized clinical trials in China revealed that various types of CHM interventions may have beneficial therapeutic effects on IPF. The number of RCTs included in this study was not large, and the intervention types were divided into four types according to whether the experimental group was added with N-acetylcysteine or conventional medical treatment. Therefore, most of the results could only be presented in the form of qualitative description. It is noteworthy that only a few studies have reported major outcomes of IPF, including acute exacerbations and mortality of IPF, making data synthesis difficult. In this review, age, gender proportions, number of patients, course of disease, syndrome differentiation of TCM, types of CHMs, dosage, and treatment course were all considered. Due to the flaws in study designs and the small sample sizes of the trials included in this review, the current evidence is insufficient to make a routine suggestion of CHMs for IPF treatment. Specially, there was very limited evidence from RCTs included in this study on the effect of CHMs on the primary efficacy outcomes of IPF. Acute exacerbation is a clinically relevant event in the course of disease in patients with IPF. It occurs in approximately 5-15% of IPF patients and is more common in advanced stage patients [53]. Respiratory infection and seasonal factors may increase the risk of acute exacerbation of IPF [54, 55]. Acute exacerbations of IPF are usually associated with lung function reduction and high morbidity and mortality [56, 57]. One study showed poor prognosis in AE-IPF patients, with in-hospital mortality exceeding 60%. And of those who survived, about 90% died within 24 weeks after discharge [56]. Therefore, controlling IPF acute exacerbations now is the main treatment purpose of many randomized controlled trials [58, 59]. Only one trial included in this review reported acute exacerbation and mortality of IPF. The analysis demonstrated that, after 72 weeks of treatment with CHMs plus NAC, there was no significant difference in the improvement of IPF acute exacerbation and mortality compared with NAC treatment alone. It is important to highlight that this study was consistent with the result of a previous systematic review that CHMs did not improve mortality in patients with IPF. However, the results of the two studies were different regarding whether CHMs could improve the acute exacerbation of IPF [25]. Considering that only one RCT reported these two primary outcomes, we were cautious about this result, and more trials are required to further verify it. Furthermore, for the other primary outcome, health-related quality of life (QOL), the data were satisfactory. IPF patients were often subjected to poor quality of life because of limited movement and breathing difficulty. Therefore, evaluating the quality of life of patients with IPF is critical [60]. The included trials in this review measured health-related quality of life using the SGRQ scores, and we indicated the potentially beneficial effects of using CHMs or combining with conventional medicine treatment on QOL in IPF patients.

Additionally, this study looked at the effects of CHMs on lung function in IPF patients, focusing on the three indicators FVC, TLC, and DLCO. We showed that CHMs treatment had a certain improvement in lung function TLC and DLCO, but the effect on FVC was not significant. More specifically, the effects of CHMs treatment alone on the improvement of DLCO and TLC in lung function were not significant, but the decrease of DLCO and TLC could be delayed after combining with conventional medicine treatment. As we have known, to some extent, 6-minute walk test (6MWT) can truly reflect the pulmonary function and the degree of dyspnea of IPF patients [61]. In this study, we found that treatment with CHMs significantly improved 6MWT. However, treatment with CHMs failed to provide benefits in terms of partial pressure of oxygen in blood (PaO_2_) in our meta-analysis. It is noteworthy to mention that there were no obvious adverse events occurring in all included trials. However, the conclusion regarding safety cannot be determined from this study due to the limited evidence provided by the eligible trials. In order to evaluate the safety of CHMs, large-scale randomized clinical trials with long-term followup are required. Additionally, in light of the included trials intention-to-treat analysis (ITT) was not performed on the cases of shedding and loss to followup, and we did this work to reduce risk of bias. After analysis, we found that the cases of shedding and loss to followup in the trials had no effect on the results of meta-analysis.

Chinese herbal medicines show promising prospects for IPF treatment, but the mechanism is still unclear. As our understanding of idiopathic pulmonary fibrosis continues to increase, studies have shown that the mechanism of TCM in the prevention and treatment of IPF is mainly to regulate cytokines and cell signal transduction pathways, inhibit the synthesis of extracellular matrix and promote its degradation, regulate oxidative stress and coagulation-fibrinolysis system, and regulate the expression of Micro-RNA [62]. This hypothesis has been supported by recent clinical trials, which has demonstrated that CHMs with the functions of activating blood circulation, removing blood stasis, tonifying qi, and nourishing Yin and tongluo have unique superiority and proper effect on improving pulmonary fibrosis [63]. Certainly, more large-scale, randomized, placebo-controlled clinical trials are needed to confirm this finding. TCM is based on its own unique principle and comprehensive theory. TCM believes that the pathogenesis of IPF is mostly qi deficiency and blood stasis, and phlegm and blood stasis obstruct the lungs, so the main therapy is to tonify qi, activate blood, and remove stasis [64]. All the 13 RCTs included in this study reported the herbal formula in the form of decoction, capsule, pilula, and granule. The prescriptions consisted mainly of herbs that tonify qi, nourish yin, activate blood, and remove stasis. The detailed herbal compositions are shown in Table 2. Among them, the leguminous plant Astragalus (Huang Qi) is the most commonly used herb with tonifying qi effects in TCM formulations for IPF. It has received attention from Chinese physicians because of its purported immunomodulating. Peach kernel (Tao Ren), Carthamus Tinctorius L. (Hong Hua), and Radix Paeoniae Rubra (Chi Shao) are the typical herbs that promote blood circulation for removing blood stasis. Ophiopogonis Radix (Mai Dong), Rehmanniae Radix (Sheng Dihuang), and Radix Pseudostellaria (Tai Zishen) are also well-known herbs that nourish yin (see Table 3). Despite the limitation that the herbal formulas covered in this review were not consistent, coadministration showed promise through improving symptoms and quality of life in patients with IPF. Future studies could develop and verify mixed herbal medicines with the most active ingredients.

This systematic review showed some limitations, which mainly stemmed from the quality of reported data. Firstly, it must be acknowledged that the methodological quality of the included trials evaluating the effect of CHMs on IPF was unsatisfactory and the quality of reporting was generally poor (see Table 4). The key problem was that insufficient information was provided to assess risk of bias. The risk of bias in RCTs included in this study was high. Only one trial bias risk was low, and it completely reported the methods of random sequence generation, randomized concealment methods, blinded implementation, and loss to followup. Other trials did not explain the randomization process they used and mention the allocation method and the blinded implementation. We had reason to infer that these were unlikely to be standard RCTs, although there was no definite evidence to substantiate this inference. Therefore, the results should be interpreted cautiously because of the unclear methodology of these trials. That is to say, these trials could not draw a definite conclusion. Secondly, although we conducted a comprehensive search, most of the included trials were published studies. There may be other unpublished studies that were not included in this review. All included trials had a small sample size, which may cause random errors for positive and negative results. The results must be treated with great caution due to the poor methodological quality and sample size of the included trials to avoid limiting the promotion of this study's conclusions. Thirdly, the treatment duration of all included studies was not long enough for end-point outcomes such as IPF-related events and mortality. Furthermore, although the treatment group of this study used the prescriptions to boost qi, invigorate blood, and unblock the collaterals, they still lacked consistency. It was difficult to conduct a meta-analysis due to clinical heterogeneity, which made the data synthesis low and the conclusion unreliable.

Taken together, although the evidence strength of this study may be weakened because of the above limitations, our findings are still valuable because of the increasing prevalence and lack of evidence of CHM interventions to treat IPF. These results suggested that further studies of CHMs in the treatment of IPF were necessary. Besides, we also achieve important implications from this study for future prospective, large registration, and high-quality clinical trials in order to get explicit evidence. Since previous clinical studies have paid little attention to morbidity, survival time, acute exacerbation, and mortality of IPF patients, these issues require further attention and research by clinicians and researchers. Future clinical trials of TCM should not only overcome the limitations presented in this study, but also be conducted strictly and carefully and it is recommended to follow the CONSORT—CHM Formulas 2017 [65].

## 5. Conclusion

In conclusion, despite certain limitations, our findings manifested that the comprehensive efficacy of oral CHMs in the treatment of IPF was better than that of oral NAC, especially in improving IPF patients' quality of life, 6-minute walk test distance, and lung function (TLC, DLOC%), and seemed to be relatively safe during the course of treatment. Due to the problems of small sample size, poor methodological quality, and large variability of Chinese herb medicines, this study held a conservative attitude towards this conclusion. In light of these considerations, additional well-designed and high-quality RCTs are awaited in the future to verify our findings and to provide beneficial clinical proposals. We hope this study will stimulate proper evaluation of CHMs.

## Figures and Tables

**Table 1:** Characteristics of the included studies.

Study ID	Sample size		Gender (male/female)		Age		Course of disease/year		Syndrome differentiation of	Intervention		Course of treatment/ weeks	Outcome
	T	C	T	C	T	C	T	C	TCM	T	C		
Chen HL 2016	13	13	8/5	9/4	60.4 ± 5.9	59.7 ± 6.3	2.1 ± 0.6	2.3 ± 0.4	NA	Yiqi Tongluo Jiedu decoction	NAC	12	④⑤
Xin DY 2016	25	24	15/10	14/10	65.68 ± 7.50	65.88 ± 7.51	0.94 ± 0.35	0.93 ± 0.44	qi deficiency and blood stasis	Erjia-xiaozheng decoction	NAC	12	③⑤
Zhao GJ 2016	24	18	15/9	13/5	58.84 ± 18.53	59.52 ± 19.35	1.97 ± 1.38	2.16 ± 1.88	deficiency of both qi and yin, phlegm and static blood obstructing lung	Fuzheng-huaxian decoction	NAC	12	③④⑤⑥⑦
Zhao GJ 2018	30	30	18/12	16/14	58	59	1.97 ± 1.38	2.16 ± 1.88	deficiency of both qi and yin, phlegm and static blood obstructing lung	Fuzheng-huaxian decoction	NAC	12	③④⑤⑦
Fan MR 2012	22	21	14/8	12/9	60.98 ± 9.22	65.12 ± 8.61	4.21 ± 1.98	3.73 ± 2.25	deficiency of both qi and yin, lung collaterals obstruction	Feixiantong decoction+ CM	NAC+ CM	12	③④⑤⑦
Fan CZ 2013	20	20	13/7	11/9	65 ± 9.46	68.1 ± 12.15	NA	NA	deficiency of both qi and yin, lung collaterals obstruction	Buxu Tongbi decoction + CM	NAC+ CM	12	③⑤⑦
Li ZH 2015	34	30	18/16	14/16	58.23 ± 8.35	59.98 ± 7.43	2.38 ± 0.45	2.87 ± 0.12	deficiency of both qi and yin, phlegm and static blood obstructing lung	Yangyin Yifei Tongluo pill+ CM	NAC+ CM	12	④⑤
An SY 2017	30	30	14/16	13/17	66.8 ± 9.3	61.5 ± 8.7	3.6 ± 0.9	3.2 ± 0.8	qi deficiency and blood stasis	Jiawei-xiayuxue decoction + NAC	NAC	24	③④⑤
Liu M 2015	18	16	NA	NA	NA	NA	NA	NA	qi deficiency and blood stasis	Xuefu-zhuyu capsule +NAC	NAC	72	①②④⑤⑥⑦
Wang QJ 2016	43	37	23/20	19/18	60.51 ± 6.82	59.23 ± 6.91	22.32 ± 8.28	20.28 ± 6.81	yang deficiency and blood stasis	Wenyang-huayu decoction +NAC	NAC	12	④
Bao XY 2014	30	30	18/12	16/14	64.90 ± 10.25	65.47 ± 12.71	4.2 ± 1.50	4.3 ± 1.47	deficiency of both qi and yin, phlegm and static blood obstructing lung	Kangxian granule +NAC+ CM	NAC+ CM	12	④⑦
Yan XX 2015	60	60	34/26	35/25	63.5 ± 1.2	63.4 ± 1.3	1.03 ± 0.1	1.04 ± 0.11	qi deficiency and blood stasis	Buyang-huanwu decoction +NAC+ CM	NAC+ CM	24	④⑦
Fan MR 2015	27	28	17/11	18/9	62.36 ± 10.5	65.68 ± 10.29	NA	NA	deficiency of pectoral qi, lung collaterals obstruction	Tongfeiluo- buzongqi decoction +NAC+ CM	NAC+ CM	12	③④⑤⑦

T: treatment group; C: control group; NA: not available; CM: conventional medicine treatment; NAC: N-acetylcysteine; outcome: ① AE-IPF; ② mortality; ③ SGRQ score; ④ lung function; ⑤ 6MWT; ⑥ PaO_2_; ⑦adverse events.

**Table 2:** Compositions of the included herbal formula.

Study ID	Herbal formula	Main composition of formula
Chen HL 2016	Yiqi Tongluo Jiedu decoction	*Astragalus 50g, Adenophorae Radix 15g, Peach kernel 10g, Andrographis paniculata (Burm. F.) Nees 15g, Radix Scutellariae 30g, Lonicera japonica Thunb. 20g, Lumbricus 10g, scorpio 5g, Psoralea corylifolia Linn. 10g, Common Yam Rhizome Winged Yan Rhizome 30g, Cornus officinalis Sieb. et Zucc.15g, Radix Glycyrrhizae Preparata 10g*

Xin DY 2016	Erjia-xiaozheng decoction	*Astragalus 20g, Radix Pseudostellariae 15g, Radix scrophulariae 12g, Turtle Shell 20g, Pangolin Scales 6g, Fritillaria thunbergii Miq. 9g, Prunella vulgaris Linn. 9g, Tree Peony Bark 15g, Pumice 15g, Radix Glycyrrhizae 9g*

Zhao GJ 2016	Fuzheng-huaxian decoction	*Astragalus 30g, Ophiopogonis Radix 24g, Schisandra chinensis (Turcz.) Baill.15g, Pseudostellariae Radix 18g, Lumbricus 24g, Radix Notoginseng 6g, Rhodiola rosea Linn. 30g, Poria cocos (Schw.) Wolf. 24g, Semen Coicis 30g, Semen Benincasae 30g, Common Coltsfoot Flower 12g, Semen Armeniacae Amarum.9g, Cornus officinalis Sieb. et Zucc.15g, Psoralea corylifolia Linn. 12g, Radix Glycyrrhizae Preparata 9g*

Zhao GJ 2018	Fuzheng-huaxian decoction	*Astragalus 30g, Ophiopogonis Radix 24g, Schisandra chinensis (Turcz.) Baill.15g, Pseudostellariae Radix 18g, Lumbricus 24g, Radix Notoginseng 6g, Rhodiola rosea Linn. 30g, Poria cocos (Schw.) Wolf. 24g, Semen Coicis 30g, Semen Benincasae 30g, Common Coltsfoot Flower 12g, Semen Armeniacae Amarum.9g, Cornus officinalis Sieb. et Zucc.15g, Psoralea corylifolia Linn. 12g, Radix Glycyrrhizae Preparata 9g*

Fan MR 2012	Feixiantong decoction	*Astragalus 30g, Rehmanniae Radix 20g, Common Burreed Rhizome 10g, Curcuma zedoaria (Christm.) Rosc. 10g, Inula japonica Thunb. 15g, Rhodiola rosea Linn. 30g, Clematis chinensis Osbeck15g, Pumice20g, Radix Glycyrrhizae 10g*

Fan CZ 2013	Buxu-tongbi decoction	*Astragalus 20g, Rehmanniae Radix 20g, Common Burreed Rhizome 10g, Curcuma zedoaria (Christm.) Rosc. 10g, Turtle Shell 10g, Inula japonica Thunb. 15g, Clematis chinensis Osbeck 15g, Pumice 20g, Radix Glycyrrhizae 10g*

Li ZH 2015	Yangyin-Yifei Tongluo pill	*Astragalus, Panax quinquefolius Linn., Ophiopogonis Radix, Peach kernel, Radix Paeoniae Rubra,Salvia miltiorrhiza Bunge, Atractylodes macrocephala Koidz., Fritillaria cirrhosa D.Don, Radix scrophulariae, Citrus maxima (Burm.) Merr. cv. Tomentosa, Saposhnikovia divaricata (Turcz.) Schischk., Glossy Privet Fruit, Tokay,Radix Glycyrrhizae*

An SY 2017	Jiawei-xiayuxue decoction	*Radix Codcnopsitis Pilosulas 15g, Ophiopogonis Radix 15g, Peach kernel 9g, Schisandra chinensis (Turcz.) Baill. 9g, Rheum officinale Bail.9g, Eupolyphaga sinensis Walker 9g, Radix Glycyrrhizae 9g*

Liu M 2015	Xuefu-zhuyu capsule	*Peach kernel, Carthamus tinctorius L.,Radix Paeoniae Rubra, Rehmanniae Radix, Ligusticum chuanxiong hort, Radix Angelicae Sinensis, Bittet Orange, Platycodon grandiflorus (Jacq.) A. DC., Bupleurum chinensis DC., Achyranthes bidentata Blume, Radix Glycyrrhizae*

Wang QJ 2016	Wenyang-huayu decoction	*Peach kernel 10g, Ligusticum chuanxiong hort 10g, Radix Rehmanniae Preparata 20g, Deerhorn Glue 10g, Ephedra Herb 6, Cinnamomum cassia Presl 4g, White Mustard Seed 10g, Rhizoma Zingiberis Preparata 10g, Radix Notoginseng 2g, Radix Angelicae Sinensis 10g, Rhodiola rosea Linn. 10g, Luffa cylindrica (L.) Roem. 10g, Radix Glycyrrhizae Preparata 10g*

Bao XY 2014	Kangxian granule	*Astragalus,Radix Pseudostellariae, Ligusticum chuanxiong hort*,*Carthamus tinctorius L., Rehmanniae Radix, Radix Angelicae Sinensis*,* Campsis grandiflora*,*Radix Scutellariae*, *Lithospermum erythrorhizon Sieb. et Zucc.*, *Trichosanthes kirilowii Maxim.*, *Fritillaria cirrhosa D. Don*, *Eupatorium japonicum Thunb. *

Yan XX 2015	Buyang Huanwu decoction	*Astragalus 30g, Peach kernel 10g, Ligusticum chuanxiong hort 10g, Radix Paeoniae Rubra 10g, Carthamus tinctorius L. 5g, Radix Aconiti Lateralis Preparata (long Fried) 30g, Radix Codcnopsitis Pilosulas 20g, Poria cocos (Schw.) Wolf. 15g, Ramulus Cinnamomi 15g, Common Yam Rhizome Winged Yan Rhizome 15g, Perilla Fruit 15g, Cornus officinalis Sieb. et Zucc. 15g, Alisma plantago-aquatica Linn. 10g, Radix Rehmanniae Preparata 10g, Dried Ginger 10g, Radix Angelicae Sinensis 10g, Chinese Eaglewood 10g, Lumbricus 10g, Pinellia ternata (Thunb.) Breit. 10g, Pericarpium Citri Reticulatae 5g, Radix Glycyrrhizae 5g*

Fan MR 2015	Tongfeiluo Buzongqi decoction	*Astragalus 30g, Common Burreed Rhizome 10g, Curcuma zedoaria (Christm.) Rosc.10g, Inula japonica Thunb.15g, Clematis chinensis Osbeck15g, Chinese Starjasmine Stem 15g, Fritillaria thunbergii Miq.20g, Polygonum cuspidatum 10g, Cimicifuga foetida Linn. 6g, Radix Glycyrrhizae 10g*

**Table 3:** Herbs frequently used and common traditional Chinese medicine diagnostic categories.

Herbal medicine	Frequency		TCM diagnosis
	Count	%	
*Astragalus *(Huang Qi)	10	76.92	Qi deficiency
*Radix Pseudostellaria *(Tai Zishen)	4	30.77	Qi and Yin deficiency
*Radix Ophiopogonis* (Mai Dong)	4	30.77	Yin deficiency
*Rehmanniae Radix* (Sheng Dihuang)	3	23.08	Yin deficiency
*Peach kernel* (Tao Ren)	6	46.15	Blood stasis
*Ligusticum chuanxiong Hort* (Chuan Xiong)	4	30.77	Blood stasis
*Carthamus tinctorius L.* (Hong Hua)	3	23.08	Blood stasis
*Radix Paeoniae Rubra* (Chi Shao)	3	23.08	Blood stasis

TCM: Traditional Chinese Medicine.

**Table 4:** Summary of finding table: Chinese Herbal Medicines compared to N- acetylcysteine for IPF.

*CHMs + NAC vs. NAC*					

*Patient or population*: [Patients with Idiopathic pulmonary fibrosis]					
*Setting*: inpatients or outpatients					
*Intervention*: [Chinese herbal medicines+ N-acetylcysteine]					
*Comparison*: [N-acetylcysteine]					

Outcome	No. of participants (studies)	Relative effect (95% CI)	Anticipated absolute effects (95% CI)		Quality of the evidence (GRADE)
			NAC	CHMs + NAC	

AE-IPF	34 (1 RCT)	*RR 0.44* (0.04 to 4.45)	-	-	*⨁⨁*◯◯ low^a,b^

Morality	34 (1 RCT)	*RR 0.30* (0.01 to 6.84)	-	-	*⨁*◯◯◯ very low^a,b^

SGRQ (6months)	60 (1 RCT)	-	The mean SGRQ(6months) was *0*	MD *3.48 lower* (6.86 lower to 0.1 lower)	*⨁⨁*◯◯ low^a,b^

6MWT (6months)	60 (1 RCT)	-	The mean 6MWT(6months) was *0*	MD *16.93 higher* (0.45 higher to 33.41 higher)	*⨁⨁*◯◯ low^a,b^

*CHMs vs. NAC*					

*Patient or population*: [Patients with Idiopathic pulmonary fibrosis]					
*Setting*: inpatients or outpatients					
*Intervention*: [Chinese herbal medicines]					
*Comparison*: [N-acetylcysteine]					

Outcome	No. of participant (studies)	Relative effect (95% CI)	Anticipated absolute effects (95% CI)		Quality of the evidence (GRADE)
			NAC	CHMs	

SGRQ	145 (3 RCTs)	-	The mean SGRQ was *0*	MD *10.87 lower* (14.3 lower to 7.44 lower)	*⨁⨁⨁*◯ moderate^a^

6MWT	171 (4 RCTs)	-	The mean 6MWT was *0*	MD *30 higher* (26.22 higher to 33.77 higher)	*⨁⨁⨁*◯ moderate^a^

*CHMs + CM vs. NAC + CM*					

*Patient or population*: [Patients with Idiopathic pulmonary fibrosis]					
*Setting*: inpatients or outpatients					
*Intervention*: [CHMs + CM]					
*Comparison*: [NAC + CM]					

Outcome	No. of participant (studies)	Relative effect (95% CI)	Anticipated absolute effects (95% CI)		Quality of the evidence (GRADE)
			NAC + CM	CHMs + CM	

SGRQ	40 (1 RCT)	-	The mean SGRQ was *0*	MD *4.36 lower* (18.5 lower to 9.78 higher)	*⨁*◯◯◯ very low^a,c^

6MWT	147 (3 RCTs)	-	The mean 6MWT was *0*	MD *85.32 higher* (56.16 higher to 114.49 higher)	*⨁*◯◯◯ very low^a,b,c,d^

*CHMs + NAC + CM vs. NAC + CM*					

*Patient or population*: [Patients with Idiopathic pulmonary fibrosis]					
*Setting*: inpatients or outpatients					
*Intervention*: [CHMs + NAC + CM]					
*Comparison*: [NAC + CM]					

Outcome	No. of participant (studies)	Relative effect (95% CI)	Anticipated absolute effects (95% CI)		Quality of the evidence (GRADE)
			NAC + CM	CHMs + NAC + CM	

SGRQ	50 (1 RCT)	-	The mean SGRQ was *0*	MD *13.11 lower* (25.46 lower to 0.76 lower)	*⨁⨁*◯◯ low^a,b^

6MWT	50 (1 RCT)	-	The mean 6MWT was *0*	MD *109.22 higher* (12.41 higher to 206.03 higher)	*⨁⨁*◯◯ low^a,b^

*∗The risk in the intervention group* (and its 95% confidence interval) is based on the assumed risk in the comparison group and the *relative effect* of the intervention (and its 95% CI).					
*CI*: Confidence interval; *RR*: Risk ratio; *MD*: Mean difference					
*GRADE Working Group grades of evidence*					
*High quality*: We are very confident that the true effect lies close to that of the estimate of the effect					
*Moderate quality*: We are moderately confident in the effect estimate: The true effect is likely to be close to the estimate of the effect, but there is a possibility that it is substantially different					
*Low quality*: Our confidence in the effect estimate is limited: The true effect may be substantially different from the estimate of the effect					
*Very low quality*: We have very little confidence in the effect estimate: The true effect is likely to be substantially different from the estimate of effect					

*Explanations*

a. No blinding.

b. High heterogeneity.

c. Wide effect interval.

d. P<0.1 in Egger's test.

## Supplementary Materials

Supplementary Materials Appendix S1. PubMed search strategy.
