# Supplementary material for: Chinese Herbal Medicines Compared with N-Acetylcysteine for the Treatment of Idiopathic Pulmonary Fibrosis: A Systematic Review of Randomized Controlled Trials
Source: Evid Based Complement Alternat Med. 2019 Jun 13;2019:5170638. doi: 10.1155/2019/5170638 (PMC6595365; doi:10.1155/2019/5170638)
Supplement: Supplementary Materials — Appendix S1. PubMed search strategy. [file 5170638.f1.docx]

**Appendix S1. PubMed search strategy**

|  |
| --- |
| 1. Traditional Chinese medicine |
| 2. Chinese medicinal |
| 3. Chinese medicine |
| 4. Chinese herbal medicine |
| 5. decoction |
| 6. Chinese patent medicine |
| 7. Chinese medicine preparation |
| 8. integration of Chinese and Western medicine |
| 9. Idiopathic pulmonary fibrosis |
| 10. pulmonary fibrosis |
| 11. pulmonary interstitial fibrosis |
| 12. Idiopathic pulmonary interstitial fibrosis |
| 13. IPF |
| 14. PF |
| 15. random* |
| 16.#1 or #2 or #3 or #4 or #5 or #6 or #7 or #8 |
| 17. #9 or #10 or #11 or #12 or #13 or #14 |
| 18. #16 and #17 |
| 19. #18 and #15 |
